# Fluoxetine effects on periodontogenesis: histomorphometrical and immunohistochemical analyses in rats

**DOI:** 10.1590/1678-77572015-0564

**Published:** 2017

**Authors:** Luciana Silva REGUEIRA, Priscylla Gonçalves Correia Leite de MARCELOS, Isabela Maria SANTIAGO-JAEGGER, Danyel Elias da Cruz PEREZ, Joaquim EVÊNCIO, Liriane BARATELLA-EVÊNCIO

**Affiliations:** 1 Universidade Federal de Pernambuco, Faculdade de Odontologia, Recife, PE, Brasil.; 2 Universidade Federal de Pernambuco, Faculdade de Odontologia, Departamento de Clínica e Odontologia Preventiva, Seção de Patologia Oral Recife, PE, Brasil.; 3 Universidade Federal de Pernambuco, Faculdade de Odontologia, Departamento de Morfologia e Fisiologia Animal, Recife, PE.; 4 Universidade Federal de Pernambuco, Faculdade de Odontologia, Departamento de Histologia e Embriologia, Recife, PE, Brasil.

**Keywords:** Periodontium, Fluoxetine, Serotonin

## Abstract

Reports have indicated that serotonin plays an important role in cell migration and differentiation during the organogenesis of several tissues, including the oral types. Administration of selective serotonin reuptake inhibitor (SSRI) drugs during pregnancy could affect the delivery of serotonin to embryonic tissues altering its development.

**Objective:**

This study aimed to assess the effects of fluoxetine, a selective serotonin reuptake inhibitor, on the formation of the periodontal ligament during pregnancy and lactation in rat pups.

**Material and Methods:**

Twelve pregnant rats of Wistar lineage were divided into four study groups. In the control group, 0.9% sodium chloride solution was administered orally, throughout the entire period of the 21 days of pregnancy (CG group) and in the CGL group, it was administrated during pregnancy and lactation (from day 1 of pregnancy to the 21st day after birth). Fluoxetine was administered orally at the dose of 20 mg/kg in a group treated during pregnancy only (FG group), and during pregnancy and lactation (FGL group). Histometrical, histochemical and immunohistochemical analysis of the maxillary first molar periodontium region of the 24 rat pups was made under light microscopy, and periodontal ligament collagen was qualitatively evaluated under a polarizing light microscope.

**Results:**

The quantity of fibroblasts (p=0.006), osteoblasts (p=0.027) and cementoblasts (p=0.001) was reduced in pups from the rats that received fluoxetine during pregnancy and lactation. No alterations were seen in the collagen fibers.

**Conclusion:**

These findings suggest that periodontal tissue may be sensitive to fluoxetine, and its interference in reducing periodontal cells depends on exposure time during lactation.

## Introduction

There have been an increasing number of prescriptions for antidepressants since 1990, particularly after the introduction of Selective Serotonin Reuptake Inhibitors (SSRIs), which are indicated in clinical situations such as depression, obsessive compulsive and bipolar disorders. In this context, fluoxetine - an SSRI, is one of the most prescribed drugs for the treatment of depressive disorders, even during pregnancy and lactation^30^. However, significant plasma levels of fluoxetine have been reported in newborns, due to its long half-life and clinically active metabolites^28^.

Blood samples from the umbilical cords obtained from newborns exposed to fluoxetine in the uterus have shown significant serum levels, equivalent to 60% of the mother’s dose^11^, which justifies and provides support for conducting research with the aim of detecting possible teratogenic effects on babies exposed to this drug. Selective serotonin reuptake inhibition (5HT), caused by the use of fluoxetine, increases the extracellular concentration of this neurotransmitter, which plays important roles in the regulation of behavior, mood, anxiety, aggressiveness, depression, sleep, fatigue and appetite suppression^6^.

Serotonin has also been described as a signal for development, due to its inductive action in tissue proliferation and morphogenesis, neuronal differentiation, cell migration, expression of growth factors, cellular and extracellular adhesion^19^.

A study^21^ investigated the pattern of 5HT receptors in the development of rat embryos and verified malformations that involved defects in the neural tube and frontonasal process arising from exposure to 5HT antagonists. The authors concluded that the use of serotonin antagonists modulates the role of this neurotransmitter in the morphogenesis of diverse craniofacial regions, and could have the same effect when these drugs are administered during pregnancy. In the same study, 5HT receptors were detected in tooth germs and the mandibular mesenchyme, in addition to the endocardium, striated muscle and neural crest.

Evidence has shown the role of serotonin on bone physiology, since it regulates osteoclast differentiation^2^ ^,^ increases the quantity of osteoprotegerin (OPG) and diminishes the osteoclast-activation factor RANKL, whereas fluoxetine causes the opposite effects^14^. Furthermore, both the osteoblasts and osteocytes of rats are capable of synthesizing 5-HT, as well as expressing functional components of serotonin receptors and transporters^4^.

Serotonin has also been reported to be a possible modulator of sensorial signals from the masticatory muscles and mechanoreceptors of the periodontal ligament^22^. The presence of serotonin in the sympathetic nerve fibers of the periodontal ligament reinforces the relationship of this neurotransmitter with the morphogenesis and homeostasis of oral structures^26^. Since the serotonin system has a regulatory function in the morphogenetic processes of neural and non-neural tissues, including enamel and dentin^10^ and as periodontal tissues have been found to be sensitive to fluoxetine^5^, it is possible that early exposure to this drug may interfere in periodontogenesis.

In view of the presence of serotonin absorption sites in embryonic dental structures^20^, the hypothesis of the interference of Selective Serotonin Reuptake Inhibitors on periodontium formation is plausible. However, there are no studies in the literature that have evaluated this hypothesis. Thus, the aim of this study was to investigate the influence of fluoxetine on the formation of the periodontium in rats whose mothers were treated with fluoxetine (20 mg/kg) during pregnancy and lactation.

## Material and methods

### Animals

A randomized, blind experimental study was conducted. The research was approved by the local Ethics Commission on the Use of Animals (protocol 23076.017680/2011-83). The experimental procedures and animal care were in accordance with the European Convention for the Protection of Vertebrate Animals used for Experimental and Other Scientific Purposes.

A total of 12 rats (*Rattus norvegicus albinus* Wistar), with an average weight of 250 g, were randomly divided into the study groups. The animals were kept in a room at a temperature of 23 +/-2°C, with a 12/12 hour light/dark cycle (light from 06:00 h to 18:00 h and dark from 18:00 h to 06:00 hours) and received the standard vivarium diet (Presence of rats and mice – Presence, Paulínia, São Paulo, Brazil) and water *ad libitum*. The adult animals were mated and daily vaginal smear tests were performed to prove pregnancy. The day on which pregnancy was proved was considered the first day of pregnancy and the beginning of the animals’ treatments.

### Treatment

Four groups of 6 pups comprised the research: the control pregnancy group (CG), which the pups came from rats that received oral administration of 0.9% sodium chloride solution during the entire period of pregnancy; control pregnancy and lactation group, in which the pregnant rats received oral administration of 0.9% sodium chloride solution during pregnancy and lactation (Group CGL); group treated with fluoxetine hydrochloride (Pharma Nostra, Rio de Janeiro, RJ, Brazil) diluted in saline to make a solution with a concentration of 20 mg/kg/ml administered orally up to the end of pregnancy (FG), and FGL group, which was treated with fluoxetine during pregnancy and through to the end of lactation, i.e., 21 days after birth. From each litter, two male pups were randomly selected, to compose the number of six animals *per* study group.

At 25 days of life, the pups of all the groups were anesthetized by an intramuscular injection of ketamine hydrochloride (50 mg/kg) and xylazine (20 mg/kg), and were perfused via the intracardiac route with a 4% formaldehyde solution. The specimens were obtained by making a frontal cut in the maxilla at a tangent to the mesial surface of the first molar, in order to analyze the periodontium of the palatine root of the maxillary first molars.

### Histological processing and morphological analyses

To characterize a blind test, each animal received an identification code attributed by a researcher who did not participate in the analysis process. The specimens were fixed in a neutral solution of 4% formaldehyde for 24 hours at room temperature. After this, they were decalcified in 5% EDTA (Vetec Química Fina Ltda, Duque de Caxias, RJ, Brazil) for 15 days. After decalcification, the specimens were submitted to conventional histological processing for inclusion in paraffin. Semi-serial histological cuts of 5 µm thickness were obtained and stained with hematoxylin-eosin (HE), Masson’s Trichrome (TM) and picrosirius red, and mounted on Entellan^®^ (Merck Millipore, Darmstadt, Hessen, Germany).

For collagen analysis, the histological slides stained with picrosirius red were observed by fluorescence emitted by the tissue, using a polarizing petrographic microscope (Olympus BX40, Olympus Corporation, Tokyo, Honshu, Japan).

For the histomorphometrical analysis of the histological sections stained with HE, a light microscope, model ECLYPSE 51^®^ (Nikon, Tokyo, Honshu, Japan), was used, coupled to a microcamera, connected to a computer with an image acquisition card (ATI) and running the IMAGE J software program for histomorphometry (SciJava software ecosystem – New York, New York, EUA). From each animal ten fields from the middle third of the palatine root were obtained, using a 20X magnification to measure the periodontal ligament thickness and quantify the osteoblasts, osteoclasts, cementoblasts and fibroblasts for each field. For the cell count *per* field, the cells considered osteoblasts were those adjacent to the axis parallel to the bone matrix; those considered osteoclasts were the multinucleated, acidophilic cells adjacent to the bone; cementoblasts were identified as being cells found parallel and adjacent to cementum, and fibroblasts as fusiform cells disposed obliquely following the direction of collagen fibers.

### Immunohistochemical analysis of type I collagen fibers

To perform the immunohistochemistry for the detection of type I collagen fibers Anti-Collagen I antibody ab34710 was used (Abcam Inc., Cambridge, Massachusetts, USA). The specimens were fixed in neutral buffered paraformaldehyde (Sigma Aldrich, São Paulo, SP, Brazil) 4% for 20 hours at 4°C. After dehydration and diaphanization by conventional procedures, the specimens were embedded in paraffin and sections of 5 μm were obtained and mounted on high grip silanized slides (EasyPath, São Paulo, SP, Brazil). Sections were deparaffinized, rehydrated, and then antigen retrieval was performed with a sodium phosphate buffer solution (PBS), pH 7.6 in a pressure cooker for 30 minutes.

After this procedure, the preparations were treated with hydrogen peroxide, 3% solution in absolute methanol for 15 minutes at room temperature to block endogenous peroxidase. Then, they were washed in PBS three times for five minutes *per* wash. The areas around the sections on the slides were cleaned thoroughly and the primary antibody was used at a dilution of 1:500 for 30 minutes, according to the manufacturer’s protocol. They were then rinsed again in PBS three times for five minutes each. They samples were then left to the addition and reaction of the “Kit” N-Histofine^®^ Simple Stain MAX PO (MULTI) (immuno-peroxidase Universal polymer, anti-mouse and rabbit - Nichirei Biosciences Inc., Tokyo, Honshu, Japan). In each section 2 drops were added to provide complete coverage of the tissues, for 30 minutes at room temperature. Again, they were washed in PBS three times for five minutes each. The diaminobenzidine chromogen was applied (DAB - EasyPath, São Paulo, SP, Brazil) to preparations to provide complete coverage of the sections for 20 minutes at room temperature. The sections were washed in distilled water 3 times for five minutes. The preparations were counterstained with Harris hematoxylin (Leica Microsystems, Nussloch, Rhein-Neckar-Kreiss, Germany) for 5 minutes, washed in water, cleared in xylene and mounted on Entellan^®^ (Merck Millipore, Darmstadt, Hessen, Germany). The preparations were observed and photographed under a light microscope, Leica ICC50 HD (Leica Microsystems Nussloch, Rhein-Neckar-Kreiss Germany).

The intensity of the staining for each antibody was determined in a semi-qualitative assessment. Immunoreactivity for collagen type I in the major tissues and cells in the periodontium was characterized by a score (0, +/-, +, ++, +++). The score ranged from negative stain (0) to a very strong positive stain (+++). A score of +/- represents tissues/cells which were weakly positive but some negative, a score of + represents tissue/cells which were positive but with weak staining and a score of ++ represents tissues/cells which were positive with strong staining.

### Statistics

The quantitative data were tabulated using the Software programs SPSS 13.0 for Windows and Excel 2007. The Kolmogorov-Smirnov Test of Normality was applied and comparisons between two groups were made using the Mann-Whitney U test, since the hypothesis of normality of the data was excluded**.** All tests were applied considering the significance level of 95% (p<0.05).

## Results

The rats had an average of 10 pups *per* litter, but only the male offspring were included in the study groups. During the experiment one FG group puppy was stillborn.

### Histological and biochemical analysis

**Figure 1:**
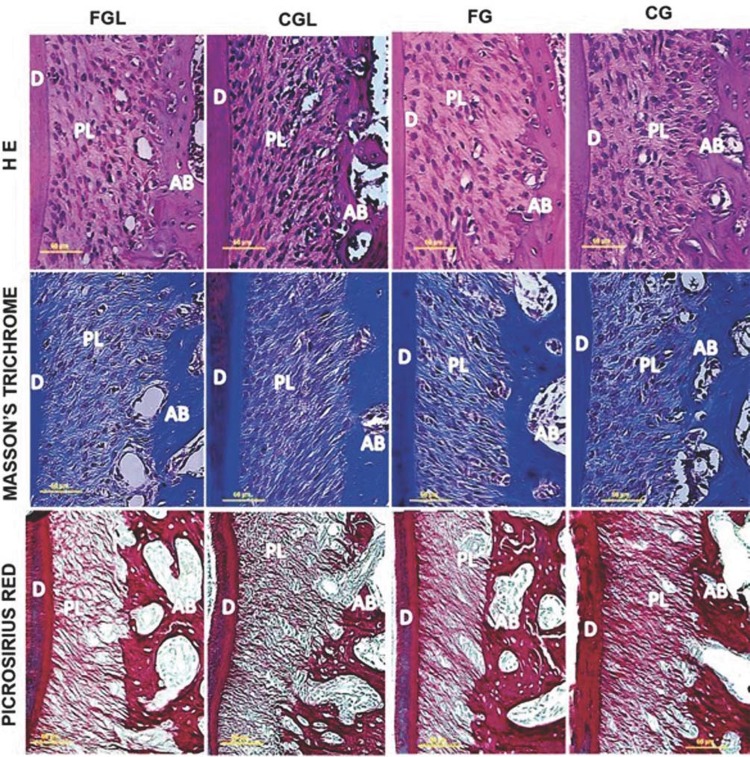
Photomicrographs of the periodontium corresponding to the middle third of the palatine root of maxillary first molars of rats at 25 days of life. Control group in Pregnancy (CG), Fluoxetine in Pregnancy (FG), Control in Pregnancy and Lactation (CGL) and Fluoxetine in Pregnancy and Lactation (FGL). Images stained with hematoxylin and eosin, Masson’s Trichrome and picrosirius red. Note the cells that compose the structures of the periodontium and periodontal ligament fiber bundles being inserted into the cementum and alveolar bone. AB: Alveolar Bone. LP: Periodontal Ligament. D: Dentin. Bar equivalent to 56 µm

The histological analysis showed no evidence of any difference in the periodontal morphology between the experimental groups (Figure 1). In all groups, the maxillary first molar was at the stage of rhizogenesis, with approximately half the root formed and the crown already having erupted. At this stage, the root apex was found to be open and a thin layer of dentin and remainders of Hertwig’s epithelial sheath were found in this region.

The periodontium in the apical region presented a large quantity of undifferentiated cells and osteoclasts, characterizing the physiological processes of tooth eruption. The cervical portion of the root showed a looser periodontal tissue due to remodeling and redirection of the periodontal fiber bundles for eruption of the tooth. Whereas, in the middle portion of the root, the conjunctive fibers were shown to be more mature and organized, showing bundles being inserted into the cementum and alveolar bone. Throughout the extension of the ligament, the collagen fibers were found to be interlaced with an intercellular substance, blood vessels, fibroblasts and fibrocytes. Cementoblasts and osteoblasts were also found adjacent to the cementum and alveolar bone surfaces, respectively. Close to the bone, osteoclasts with normal morphology were also observed.

In all groups, the trabecular alveolar bone was shown to be thin, discontinuous and with ample medullary spaces. Osteoblasts were observed at the margin of the alveolar bone, as well as osteocytes engulfed within the trabecular bone, showing cortical bone in development. With further reference to the bone structures, the presence of blood vessels belonging to the Haversian system was observed in the supporting alveolar bone.

Cementum deposition was observed in the form of a thin, acellular layer, accompanying the process of dentinogenesis on root dentin. Cementoblasts were shown to be oval, with the long axis parallel to the root surface.

Both the alveolar bone and cementum presented a strong red color in the histological sections stained with picrosirius red. In the peripheral portions of the periodontal ligament, the presence of mature collagen fibers was verified, and therefore, with a more intense red staining with reference to the Sharpey fibers. The collagen fiber bundles of the central portion of the ligament were shown to be looser and immature, evidenced in Masson’s Trichrome by a light blue coloring and in picrosirius red by less intense red staining.

### Polarizing microscopy analysis

**Figure 2:**
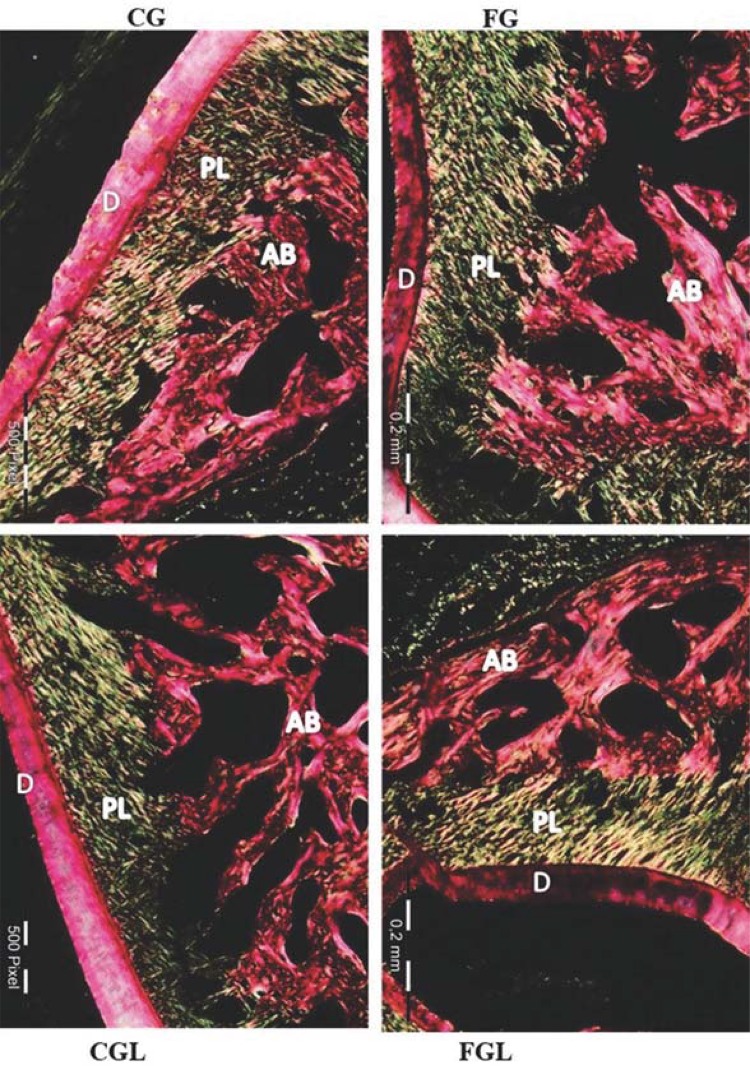
Photomicrographs of the periodontium corresponding to the middle third of the palatine root of maxillary first molars of rats at 25 days of life. Control group in Pregnancy (CG), Fluoxetine in Pregnancy (FG), Control in Pregnancy and Lactation (CGL) and Fluoxetine in Pregnancy and Lactation (FGL). Cuts stained with picrosirius red and analyzed under a polarizing microscope. Note the predominance of colors green and yellow in periodontal ligament collagen fibers, indicating the presence of Type III and Type I collagen, respectively. In alveolar bone, the predominance of type I collagen was verified (red coloring). AB: Alveolar Bone. LP: Periodontal Ligament. D: Dentin. Bar equivalent to 0.2 mm

The samples evaluated presented no alterations as regards the type and disposition of collagen in the periodontium of animals that were either submitted to fluoxetine, or not, during their development (Figure 2). Under polarizing microscopy analysis, picrosirius red showed coherence of periodontal ligament collagen organization in both yellow and green colors in both experimental groups. The presence of collagen was also observed in the basal lamina and acellular cementum, reflecting a green and yellow color. The alveolar bone presented a predominance of red collagen fiber in all study groups.

The polarization technique allowed for the evaluation of the direction and type of collagen fiber bundles, which were disposed obliquely and ascending in the cement-alveolar bone direction.

### Histomorphometric analysis

The measurements performed revealed no statistical difference between the CG and FG groups, for any of the analyzed variables (Table 1).

**Table 1:** Evaluation of periodontal ligament thickness and quantity of periodontium cell type in Groups CG and FG

Variables	Group CG	Group FG	p-Value*
	Mean + SD	Mean + SD	
Periodontal Ligament thickness	290.17i ± 26.68i	287.03 ± 21.45	0.447
Fibroblast	57.35 ± 13.86	61.88 ± 9.66	0.091
Osteoblast	8.28 ± 2.69	7.23 ± 2.23	0.081
Osteoclast	0.67 ± 0.77	0.45 ± 0.65	0.105
Cementoblast	9.15 ± 2.91	10.23 ± 2.81	0.073

(*) Mann-Whitney Test – P<0.05

(i) inches

In the animals submitted to fluoxetine during pregnancy and lactation, alterations were identified in the number of cells of which the periodontium is composed. The FGL group showed a statistically significant reduction in the quantity of fibroblasts (p=0.006), osteoblasts (p=0.027) and cementoblasts (p=0.001) when compared with its respective control group (CGL). The measurements of periodontal ligament thickness and quantity of osteoclast between the CGL and FGL groups showed no statistically significant differences (Table 2).

**Table 2:** Evaluation of periodontal ligament thickness and quantity of periodontium cell types in the CG and FG groups

Variables	Group CGL	Group FGL	p-Value*
	Mean + SD	Mean + SD	
Periodontal Ligament thickness	287.21i ± 29.63i	294.04 ± 37.93	0.445
Fibroblast	58.68 ± 13.98	51.68 ± 11.66	0.006
Osteoblast	7.30 ± 2.47	6.33 ± 2.43	0.027
Osteoclast	0.40 ± 0.56	0.32 ± 0.57	0.292
Cementoblast	10.95 ± 2.09	9.75 ± 2.49	0.001

(*) Mann-Whitney Test – P<0.05

(i) inches

### Immunohistochemical analysis of collagen I

**Figure 3:**
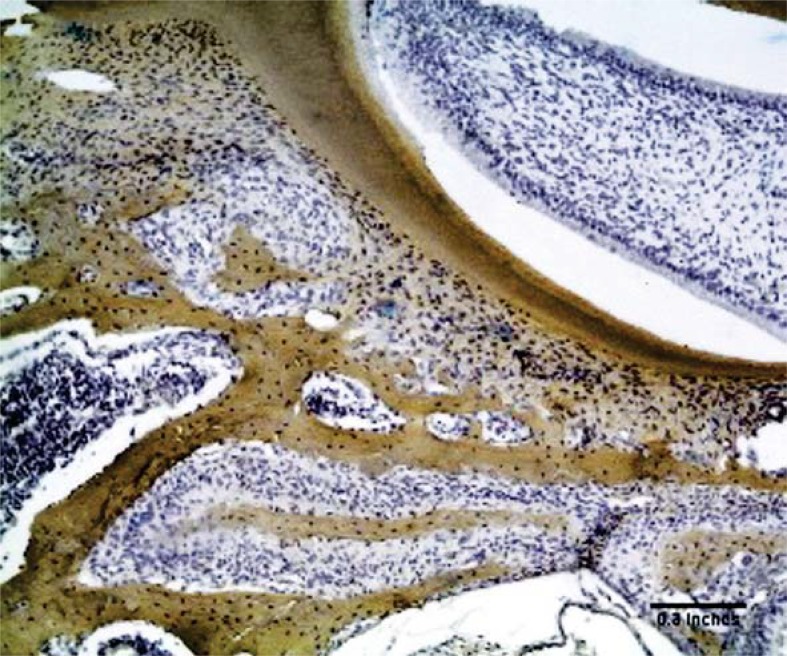
Control group – Periodontal ligament presenting moderate positivity for collagen type I. Bar equivalent to 123 µm

Type I collagen immunoreactivity was evident by a very strong positive stain (+++) in the alveolar bone and dentin in all groups. The periodontal ligament showed a positive but weak staining in all specimens (Figure 3).

## Discussion

In the present study, the histological findings relative to the stage of tooth development and the morphology of structures present in the periodontium of the animals were in conformity with the description of the rat periodontium in previous studies thus conferring a pattern of morphological normality in the experimental groups^1^.

The histomorphometrical analysis revealed a significant reduction in the number of cementoblasts, osteoblasts and fibroblasts in the periodontium of pups from the FGL group, whose mothers received fluoxetine during pregnancy and lactation. However, there were no alterations in the group of animals exposed to fluoxetine during pregnancy only (FG group).

It has been reported that the effects of the use of fluoxetine during lactation can be transmitted to the newborn^25^. The clearance of fluoxetine decreases in the postpartum period and fluoxetine serum in the mother is higher in this period than in the third trimester of pregnancy^18^, which could explain the fibroblast, cementoblast and osteoblast reduction in the periodontal ligament in only the FGL group.

Although the direct interference of fluoxetine in the cells of the periodontium has not been reported in the literature, some studies have reinforced the foundation for this hypothesis. The tooth follicle, responsible for the formation of the periodontium, is derived from the neural crest cells^27^. Serotonin is responsible for regulating cell migration from the neural crest during neurulation in a dose-dependent manner^23^. Therefore, the effects of selective serotonin reuptake may affect structures such as the periodontium, derived from this annex.

Studies previously conducted by our research group did not reveal alterations in the formation and morphology of the temporomandibular joint (TMJ)^7^ and tooth enamel of rat pups treated with fluoxetine (10 mg/kg) during pregnancy^29^. Nevertheless, the administration of 20 mg/kg fluoxetine during rat pregnancy and lactation modified the jawbone mass of the pups^8^. Taking these results into consideration, besides the fact that the dose most used in humans is 20 mg/kg, we opted to conduct our study using the 20 mg/kg dose. The alterations detected at the dose of 20 mg/kg can be explained by the possibility of fluoxetine’s role on periodontogenesis following the dose-dependent pattern described by Moiseiwitsch and Lauder^24^ (1996) when they analyzed the development of the tooth germ *in vitro*.

Hodge, et al.^15^ (2012) demonstrated the involvement of fluoxetine in bone metabolism in an *in vitro* study. The authors detected that both osteoblasts and osteoclasts, in addition to expressing the serotonin transporter (5-HTT) and the serotonin receptor 5-HTR1B, also presented tryptophan hydroxylase, an enzyme that catalyzes tryptophan for the formation of serotonin. In this same study, fluoxetine was shown to inhibit the quantity of osteoclasts and resorption, in a dose-dependent manner. At a high dose, fluoxetine also inhibited mineralization by the osteoblasts, but did not alter the morphological parameter of the cells in culture. Whereas, in the present study, no significant alterations were found in the number of osteoclasts.

There is convincing evidence that serotonin modulators such as SSRIs can affect bone turnover due to the capacity of the 5-HT2BR receptor to facilitate osteoblast recruitment and proliferation^9^ ^,^ ^13^. With regards to fibroblasts, it has been established that these cells express chains of intercellular signals activated by the serotonin receptor 5HTlc^16^. There are no studies that correlate the effects of serotonin and its inhibitors on cementoblasts. However, as these cells originate from the same embryonic tissue as fibroblasts and osteoblasts, the tooth follicle, it is suggested that these cells are modulated in a similar manner by serotonergic stimuli.

Type I collagen is the largest component of the periodontal ligament^17^. Procollagen and type III collagen are components of the periodontal ligament fiber bundles and their colocation with type I collagen in the same fiber has been suggested^3^. In the present study, the types of collagen fibers found in the periodontal ligament did not present differences between both experimental groups observed under immunohistochemical analyses, what is in conformity with the description of the periodontium reported in the literature^12^.

## Conclusion

The results obtained suggested that periodontal cells are sensitive to the action of fluoxetine and it appears to be dependent on the time of exposure, since only the group exposed during pregnancy and lactation presented a decrease in the quantities of fibroblasts, cementoblasts and osteoblasts. Despite the cell reduction in the FGL group, the descriptive morphological analysis showed no structural periodontal changes, so further studies must be conducted to confirm the involvement of fluoxetine on periodontogenesis.
